# Exploring the Mediating Role of Sleep Deficit-Related Functional Status in Subacute Stroke Survivors

**DOI:** 10.3390/medicina60030422

**Published:** 2024-02-29

**Authors:** Sunil Kumar, Sarah Parveen, Md Dilshad Manzar, Ahmad H. Alghadir, Masood Khan, Khalid Wasel Al-Quliti, David Warren Spence, Seithikurippu R. Pandi-Perumal, Ahmed S. Bahammam, Majumi M. Noohu

**Affiliations:** 1 Center for Physiotherapy and Rehabilitation Sciences, Jamia Millia Islamia, New Delhi 110025, India; drsuniljmi@gmail.com (S.K.); sarahjmi880281@gmail.com (S.P.); mnoohu@jmi.ac.in (M.M.N.); 2 Department of Nursing, College of Applied Medical Sciences, Majmaah University, Majmaah 11952, Saudi Arabia; m.manzar@mu.edu.sa; 3 Department of Rehabilitation Sciences, College of Applied Medical Sciences, King Saud University, Riyadh 11433, Saudi Arabia; aalghadir@hotmail.com; 4 Department of Medicine, College of Medicine, Taibah University, Madinah 42353, Saudi Arabia; kh_alquliti@yahoo.com; 5 Independent Researcher, 652 Dufferin Street, Toronto, ON M6K 2b4, Canada; dwspence@fastmail.fm; 6 Saveetha Medical College and Hospitals, Saveetha Institute of Medical and Technical Sciences, Saveetha University, Chennai 600077, India; pandiperumal2023@gmail.com; 7 Division of Research and Development, Lovely Professional University, Phagwara 144411, India; 8 Department of Medicine, College of Medicine, King Saud University, Riyadh 11461, Saudi Arabia; ashammam2@gmail.com

**Keywords:** post-stroke fatigue, sleep, stroke, functions

## Abstract

*Background:* Understanding post-stroke fatigue (PSF) and its associated factors is crucial for effective therapy and rehabilitation. This study aimed to assess the mediating role of the excessive daytime sleepiness-related functional status (SFS) on the relationship between sleep and the severity of fatigue in subacute stroke survivors. *Methods:* Subacute stroke survivors (n = 50; male = 38; female = 12), completed a cross-sectional study involving the Pittsburgh sleep quality index (PSQI), the Epworth sleepiness scale (ESS), the insomnia severity index (ISI), the functional outcome of the sleep questionnaire (FOSQ), and the fatigue severity scale (FSS). *Results:* The SFS mediated the association between the severity of fatigue and sleep problems. The PSQI and FOSQ (b = −0.37, *p* < 0.001), and the FOSQ and FSS were correlated (b = −0.18, *p* < 0.05), with a significant indirect effect of the PSQI on the FSS. The ISI correlated with the FOSQ (b = −0.20, *p* < 0.001), with significant direct (b = 0.15, *p* < 0.001), as well as indirect, effects of the ISI on the FSS. The ESS correlated with the FOSQ (b = −0.23, *p* < 0.001), with a significant indirect effect of the ESS on the FSS. *Conclusions:* In subacute stroke survivors, fatigue and sleep are linked. Increased understanding of sleep-PSF may help in exploring new targets for supplement therapy.

## 1. Background

Sleep is a critical component in different physiological functions such as cognition, mental health, immune responses, and physical activity [1,2]. Sleep deprivation negatively affects body functions [3]. The commonly reported sleep problems among the otherwise healthy population are insomnia, poor sleep quality, and excessive daytime sleepiness. The aforementioned sleep problem is usually identified under the umbrella term sleep disorder [4,5]. People with stroke have also been identified as having sleep disorders. The reason might be the damage to the different neurological structures regulating normal sleep physiology. The areas of the brain that are specifically attributed to controlling sleep–wake cycles are the brainstem, midbrain, and thalamus [6]. A very high prevalence (78%) of sleep disorders has been reported in acute stroke. The reported pooled prevalence of insomnia was 38%, while the excessive daytime sleepiness ranged from 18 to 72% [5,7].

Post-stroke fatigue (PSF), along with residual neurological deficits, is recognized as a significant concern among stroke survivors. Fatigue in a healthy population and PSF have similar definitions. It is described as a feeling of early exhaustion with weariness, lack of energy, and aversion to the effort that develops during physical or mental activity and is usually not alleviated by rest [8]. The mechanism behind PSF is still not well established. The various reasons suggested for post-stroke fatigue include increased tissue inflammation [9], alterations in excitability, and disruptions in the connections between various brain areas due to stroke pathology [10]. Central models of fatigue attribute this condition to changes in the functioning of the CNS [11]. The reported prevalence of PSF is around 50%, and it may improve or worsen over time [12,13]. The presence of PSF can affect the quality of life, treatment, and rehabilitation outcomes [14,15]. Sleep disturbances and fatigue are commonly associated in individuals with stroke, potentially due to alterations in brain function in this population. Studies have shown that the presence of sleep disorders has a negative impact on functional outcomes following stroke. However, the excessive daytime sleepiness-related functional status is not specifically reported [16,17].

It is imperative to explore the factors that may mediate or influence the relationship of factors leading to specific outcomes [18,19]. There may be different factors that may affect the relationship of fatigue and sleep [20]. There is a lack of information on how the excessive daytime sleepiness-related functional status mediates the relationship between sleep quality, insomnia, daytime sleepiness, and PSF in the stroke population. The excessive daytime sleepiness-related functional status may affect daily activities, depending on both the quantity and quality of sleep [21,22]. We hypothesized that excessive daytime sleepiness-related functional status might impact the relationship between sleep measures and fatigue severity in patients with subacute stroke. The findings could clarify how the excessive daytime sleepiness-related functional status influences the relationship between fatigue and sleep parameters. This insight may aid in creating intervention strategies for these challenges within this population. Additionally, a deeper understanding of PSF will enable clinicians to more frequently recognize and effectively communicate this ‘invisible gap’ to their patients.

## 2. Methods

### 2.1. Participants, Design, and Setting

**Figure 1:**
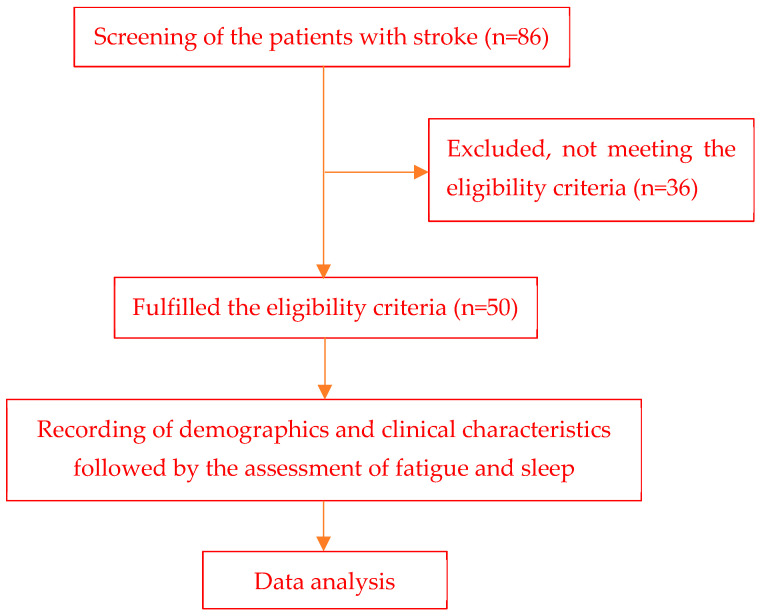
Study flow diagram of the patients with stroke.

This cross-sectional study included 50 individuals after obtaining approval from the Institutional Ethics Committee, Jamia Millia Islamia, New Delhi, India. Patients were included if they were >18 years of age, it was the patient’s first stroke, they had an MMSE score of ≥ 24, and a stroke duration of fewer than 6 months. Stroke patients with speech difficulties (aphasia), psychiatric disorders such as schizophrenia, behavioral, or affective disorders, a history of depression, severe cognitive impairment, or dementia, a history of cerebrovascular accidents, multiple or bilateral stroke lesions, who had previously been treated for sleep problems, and had a stroke duration of less than one month were excluded from the study. Eighty-six subjects were screened for the study (Figure 1). The subjects were recruited from Dr. M. A. Ansari Health Centre & Centre for Physiotherapy and Rehabilitation Sciences and the nearby locality of Jamia Millia Islamia (JMI), New Delhi, India. Medical records were used to screen the patients’ suitability for study participation.

### 2.2. Study Protocol

The individuals were pre-screened to ensure they were eligible to participate in the study. The age, height, weight, systolic blood pressure, diastolic blood pressure, comorbidities, and stroke history of each participant were recorded. This was followed by an evaluation of sleep and fatigue using standardized questionnaires. The session lasted between 1 and 1.5 h and was conducted by a professional with over five years of experience in treating or assisting in the treatment of patients with neurological disorders.

### 2.3. Measures

#### 2.3.1. Fatigue Severity Scale (FSS)

The fatigue severity scale (FSS) is a self-administered questionnaire with nine items (questions) that assess fatigue severity by quantified ratings of its occurrence in various circumstances throughout the last week. Each item is graded on a scale of 1 to 7, with 1 denoting significant disagreement and 7 denoting strong agreement. The final score is the mean of the nine items [23]. It is one of the most commonly used measures for assessing fatigue in stroke patients [24]. The cut-off value of FSS for stroke patients was 3.90 ± 1.85 [25].

#### 2.3.2. Epworth Sleepiness Scale (ESS)

The Epworth sleepiness scale (ESS), comprising eight self-rated questions scored from 0 to 3, assesses excessive daytime sleepiness by measuring an individual’s tendency to fall asleep in daily situations. Scores range from 0 to 24, with higher scores indicating greater sleepiness. A score above 10 suggests significant sleepiness, while a score above 6 indicates moderate sleepiness [26]. The ESS has been shown to have adequate measures of internal reliability, internal homogeneity, item analysis, structural validity, and known-group validity in university students [27].

#### 2.3.3. Insomnia Severity Index (ISI)

This self-reported questionnaire [28,29] was used to evaluate the nature, impact, and severity of insomnia, typically recalling the previous month. It assessed several aspects of sleep, including the noticeability of sleep problems to others, difficulty maintaining sleep, the severity of sleep onset issues, distress caused by sleep difficulties, how these difficulties interfere with daytime functioning, problems with early morning awakenings, and overall dissatisfaction with sleep.

Each question is rated on a 5-point Likert scale (0 = no problem; 4 = very severe problem), resulting in a total score that ranges from 0 to 28. The following categories of insomnia were used to interpret the overall score: severe insomnia (22–28), moderate insomnia (15–21), sub-threshold insomnia (8–14), and no insomnia (0–7) [28]. The validity of the ISI has been established in various populations using robust methodologies, including Southeast Asians [30,31,32].

#### 2.3.4. Pittsburg Sleep Quality Index (PSQI)

The Pittsburg sleep quality index (PSQI), a 19-item self-rated questionnaire for assessing subjective sleep quality during the past month, was used to gauge sleep quality. Seven clinically derived component scores, each weighted equally from 0 to 3, were created from the 19 questions. A total score ranging from 0 to 21 was calculated by adding the 7 component scores, with higher numbers indicating progressively poorer sleep quality [19].

#### 2.3.5. Functional Outcomes of Sleep Questionnaire (FOSQ)

The functional outcomes of sleep questionnaire (FOSQ) measures the extent to which severe sleep disorders impact the physical, mental, and social functions necessary for daily life. It aims to assess the difficulty respondents experience in their regular activities due to tiredness or drowsiness. The questionnaire includes 30 questions across five subscales: general productivity, activity level, intimacy/sexual relationships, vigilance, and social outcome. The study utilized the short version of the FOSQ [33,34]. Questions are answered on a scale ranging from extreme difficulty to no difficulty. The sum of participants’ responses provides overall and subscale scores.

### 2.4. Statistical Analysis

Data analysis was carried out using SPSS version 21.0. The demographic and clinical characteristics were reported as the mean ± standard deviation or as frequencies/percentages. The correlation between fatigue and sleep was performed using Spearman’s two-tailed test. This study implemented a mediation model approach by Zhao et al. [35]. This approach employs a modified version of the mediation effect model proposed by Baron and Kelly [35,36]. A three-mediation model analysis with a unique mediator, the excessive daytime sleepiness-related functional status, was performed on the relationship of (i) the sleep quality and the severity of fatigue; (ii) the severity of insomnia, and the severity of fatigue; (iii) and the daytime sleepiness and the severity of fatigue, using SPSS PROCESS version 4.0 [37]. The total scores of FOSQ, PSQI, ISI, ESS, and FSS were used as measures of the excessive daytime sleepiness-related functional status, sleep quality, the severity of insomnia, daytime sleepiness, and the severity of fatigue, and sleep apnea index, respectively. In Model 1, sleep quality (PSQI score) was the IV, sleep deficit-related functional loss (FOSQ score) was the MV, and the severity of fatigue (FSS score) was the DV. In Model 2, the severity of insomnia (ISI score) was the IV, sleep deficit-related functional loss (FOSQ score) was the MV, and the severity of fatigue (FSS score) was the DV. In Model 3, daytime sleepiness (ESS score) was the IV, sleep deficit-related functional loss (FOSQ score) was the MV, and the severity of fatigue (FSS score) was the DV. The respective sub-scores of the scales were treated as continuous variables when deriving the total score. The significance of the indirect effect was determined by the bootstrap test [38]. The number of bootstrap samples was 5000 with a fixed seeding of 5024. In this test, an absence of zero from the confidence interval of coefficients indicates significance. The selection of the dependent variable (DV), independent variable (IV), and mediator variable (MV) was based on theoretical considerations and previous evidence of the relationship among these (DV, IV, and MV) variables [14,17,39]. Sleep disturbances, stroke, and sleep are associated with each other [17]. Sleep disturbances such as poor sleep quality, insomnia, sleepiness, and periodic limb movement are common in stroke survivors, affecting their functional outcome [17]. PSF is associated with sleep disturbances [39] and functional outcomes [14]. In a mediation model, the selection of covariates is made based on theoretical consideration from previous works that showed the association of some variables (prospective covariates) with all/any of three variables in the model, i.e., DV, IV, and MV, and statistical evidence based on the present data. However, we did not include covariates in this study’s analysis plan because of the small size, parsimony, as well as power considerations, and giving priority to the key variables in the model.

## 3. Results

The demographic and clinical characteristics of the participants are given in Table 1. Descriptive statistics for the presence of fatigue, sleep disturbances, and obstructive sleep apnea are presented in Table 2. The correlation analysis revealed that the FOSQ, PSQI, ISI, and FSS scores were significantly correlated with fatigue severity (Table 3).

**Figure 2:**
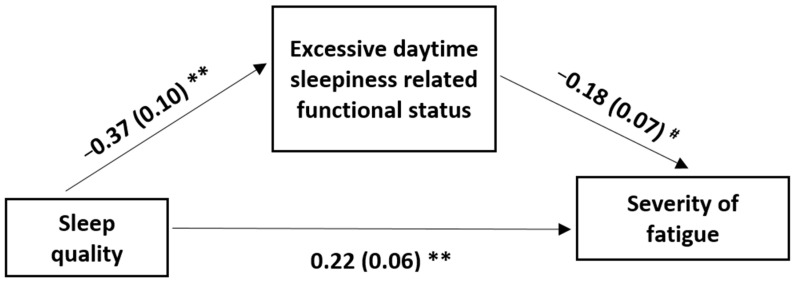
The model with sleep deficit-related functional loss (FOSQ score) as a mediator in the effect of sleep quality (PSQI score) on the severity of fatigue (FSS score). FOSQ: functional outcomes of sleep questionnaire; PSQI: Pittsburg sleep quality index; FSS: fatigue severity scale. The first values are the unstandardized coefficients, and the second values within brackets are the standard errors; ** *p* < 0.001; ^#^ *p* < 0.05. The indirect effect of sleep quality on the severity of fatigue through sleep deficit-related functional loss was significant (95% CI 0.07 [0.01, 0.13]).

Figure 2 and Table 4 show the outcome of the mediation analysis. This analysis evaluated the mediating effect of sleep deficit-related functional loss in the relationship between sleep quality and fatigue severity in stroke patients. The mediation model explained 43.64% of the variance in sleep quality (R^2^ = 0.4364 F [1, 47] = 18.19, *p* < 0.001). The PSQI score significantly and negatively correlated with the FOSQ score (β = −0.37, standard error (SE) = 0.10, *p* < 0.001), indicating that those with a poor sleep quality were likely to have more severe functional losses due to sleep deficits. Additionally, the FOSQ scores significantly and negatively correlated with the FSS scores (β = −0.18, SE = 0.07, *p* < 0.05), indicating that those with sleep deficit-related functional losses may also have more severe fatigue levels. The direct effect of sleep quality on fatigue severity stayed positively significant after adjusting for the effect of the FOSQ score (β = 0.22, SE = 0.06, *p* <0.001). The indirect effect was significant: 95% CI = 0.07 (0.01 to 0.13), indicating that sleep deficit-related functional loss was a significant mediator in the relationship between sleep quality and fatigue severity in stroke patients (Table 4).

**Figure 3:**
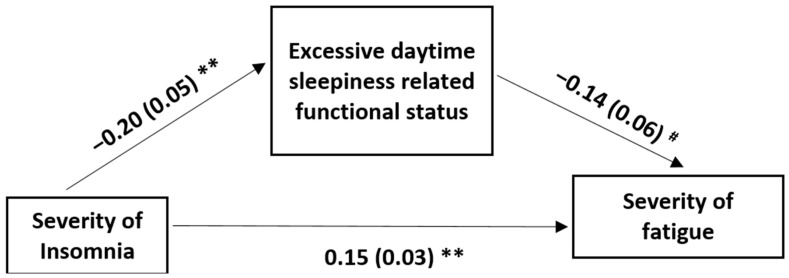
The model with the sleep deficit-related functional loss (FOSQ score) as a mediator in the effect of the severity of insomnia (ISI score) on the severity of fatigue (FSS score). FOSQ: functional outcomes of sleep questionnaire; ISI: insomnia severity index; FSS: fatigue severity scale. The first values are the unstandardized coefficients, and the second values within brackets are the standard errors; ** *p* < 0.001; ^#^ *p* < 0.05. The indirect effect of sleep quality on the severity of fatigue through sleep deficit-related functional loss was significant (95% CI 0.03 [0.002, 0.06]).

Figure 3 and Table 5 show the outcome of the mediation analysis for evaluating the mediation effect of the sleep deficit-related functional loss in the relationship between the severity of insomnia and the severity of fatigue in stroke patients. The mediation model explained 55.54% of the variance in the severity of insomnia (R^2^ = 0.5554 F [1, 47] = 29.36, *p* < 0.001). The ISI score significantly and negatively correlated with the FOSQ score (b = −0.20, SE = 0.05, *p* < 0.001), indicating that those with severe insomnia were likely to have a greater sleep deficit-related functional loss. The direct effect of the severity of insomnia on the severity of fatigue stayed positively significant after adjusting for the effect of the FOSQ score (b = 0.15, SE = 0.03, *p* < 0.001). The indirect effect was significant: 95% CI = 0.03 (0.002 to 0.06), indicating that sleep deficit-related functional losses were a significant mediator in the relationship between the severity of insomnia and the severity of fatigue in stroke patients (Table 5).

**Figure 4:**
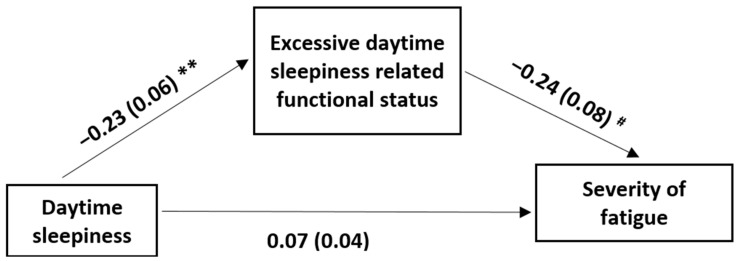
The model with sleep deficit-related functional loss (FOSQ score) as a mediator in the effect of daytime sleepiness (ESS score) on the severity of fatigue (FSS score). FOSQ: functional outcomes of sleep questionnaire; ESS: Epworth sleepiness scale; FSS: fatigue severity scale. The first values are the unstandardized coefficients and the second values within brackets are the standard errors; ** *p <* 0.001; ^#^ *p* < 0.05. The indirect effect of daytime sleepiness on the severity of fatigue through sleep deficit-related functional loss was significant (95% confidence interval 0.05 [0.01, 0.10]).

Figure 4 and Table 6 show the outcome of the mediation analysis for evaluating the mediation effect of the sleep deficit-related functional loss in the relationship between daytime sleepiness and the severity of fatigue in stroke patients. The mediation model explained 31.11% of the variance in daytime sleepiness (R^2^ = 0.3111 F [1, 47] = 10.61, *p* < 0.001). The ESS score was a significant and negative correlate of the FOSQ score (b = −0.23, SE = 0.06, *p* < 0.001), indicating that those with excessive daytime sleepiness were likely to have more sleep deficit-related functional losses. The direct effect of daytime sleepiness on fatigue severity was not significant after adjusting for the effect of the FOSQ score (b = 0.07, SE = 0.04, *p* = 0.09). However, the indirect effect was significant: 95% CI = 0.05 (0.01 to 0.10), indicating that the sleep deficit-related functional losses were a significant mediator in the relationship between daytime sleepiness and fatigue severity in stroke patients (Table 6).

## 4. Discussion

The results revealed that fatigue severity is affected by sleep problems. The relationship between fatigue severity and sleep problems may be mediated by excessive daytime sleepiness-related functional status. Sleep quality and insomnia were also contributing factors to the fatigue severity. The sleep-related functional losses were an intermediary variable that could influence the severity of fatigue.

According to the mean scores from the sleep questionnaires, respondents’ sleep deficits were associated with poor sleep quality, subthreshold insomnia, mild excessive daytime sleepiness, and reduced functional capability. The FSS mean score was 3.89, which was slightly below the cut-off score of 4 for problematic fatigue; the results are in agreement with previous reports. In one study of ischemic stroke patients, 56.7% reported sleep complaints, and 37.5% met the DSM-IV criteria for insomnia [40]. However, in other studies, the stroke population was of a different duration; they were either acute [41] or chronic [42].

Fatigue and sleep issues are also frequently encountered in those who have had a subacute stroke [43]. In the present study, patients who slept for 5–6 h scored higher on fatigue measures than those who slept for 6–7 h. Similar results were found in a study conducted by Goldman and colleagues (2008), where community-dwelling older individuals who slept less than 6 h/night had a 4.3% higher fatigue score than those who slept 7 h/night, indicating that both short and long sleep durations were related to fatigue [44]. In addition, the association between fatigue and sleep disturbances was reported in community-dwelling older adults [44], older individuals with osteoarthritis [45], patients with traumatic brain injury [46,47], and patients with multiple sclerosis [48].

Although there is evidence that a link exists between fatigue and sleep, it is unclear how this link should be interpreted. Both fatigue and sleep may be due to the same causes, such as decreased physical functioning in stroke patients. Nonetheless, this link highlights the importance of addressing sleep and fatigue as parts of a larger post-stroke problem. Studies have documented inter-relationships between PSF and sleep quality and sleep disturbances [49,50], but the mechanism behind this inter-relationship remains unclear. A relationship has been observed between poor sleep quality, excessive sleepiness, insomnia, and functional impairments in patients with mild acute stroke [21]. This particular finding is important since subjects in the present study also reported sleep deficit-related functional problems. The findings of this study suggest that the functional status related to excessive daytime sleepiness may mediate the relationship between sleep quality and PSF. The lack of clarity regarding the direction of causality and the possible role of feedback mechanisms suggests that the association variable under investigation is complex.

Sleep patterns are heritable. Mendelian randomization analysis indicates that ischemic stroke is linked with genetic liability for a small duration of sleep and frequent insomnia. Among the putative genetic links are variants of circadian genes, cardiovascular risk factors, genetic factors related to specific sleep disorders, and genes encoding parts of the neurotransmitter network [51].

Restoring mental and physical functionality among subacute stroke patients depends on rehabilitation therapy. Healthcare providers must be vigilant for problems related to fatigue and sleep in patients’ acute and subacute periods while designing and executing rehabilitation interventions. The findings of the present study, as well as those of others, support the conclusion that fatigue and sleep problems may be important barriers to recovery in subacute stroke patients. To tailor the rehabilitation program to patients’ levels of fatigue, special measures such as graded activity should be utilized.

The findings of this study have significant practical implications for clinicians and rehabilitation professionals, revealing a relationship between sleep and fatigue. This insight will not only enhance clinicians’ understanding of this relationship but also aid in developing intervention strategies for managing fatigue and sleep issues in stroke patients. Among the study’s limitations are the fact that the sample size was small and did not measure pre-stroke fatigue. Secondly, because the study did not attempt to evaluate the presence of psychological depression, it could not document the impact of this variable on sleep and fatigue measurements. The central sleep apnea evaluation was also not performed. Furthermore, this study did not examine the neuropathology, including the location and type of stroke, which could have influenced the measured parameters. Additionally, the lack of objective sleep disorder assessments, such as polysomnography, in patients with PSF represents another limitation of the study findings. Future research may focus on the influence of sleep management techniques in stroke and how they affect the mediating roles of pre-stroke fatigue and comorbidities in PSF. The relationship between fatigue and sleep parameters warrants investigation across various age groups, including older and very old adults. While exploring mediation effects with cross-sectional data lacks the statistical robustness needed for causal conclusions, implementing a longitudinal design in stroke patients may raise ethical concerns. Despite these limitations, this study’s findings offer valuable baseline data on the relationship between excessive daytime sleepiness-related functional status, sleep metrics, and fatigue severity in subacute stroke patients.

## 5. Conclusions

Fatigue and sleep disturbances are interconnected in patients experiencing subacute stroke. Therefore, therapeutic strategies for this population should emphasize comprehensive assessment, tailored interventions, and management of both sleep issues and post-stroke fatigue.

## Figures and Tables

**Table 1:** Demographic and clinical characteristics of the participants (n = 50).

Variables	Mean ± SD
Age (years)	51.840 ± 7.03
Gender (M/F)	38/12
Height (cm)	167.44 ± 6.70
Weight (kg)	70.620 ± 8.13
BMI (kg/m^2^)	25.210 ± 2.73
SBP (mmHg)	134.800 ± 10.73
DBP (mmHg)	82.900 ± 5.35
MMSE score	26.740 ± 1.84
Type of stroke (n, n%)	
Ischemic	39 (78)
Hemorrhagic	11 (22)
Type of stroke syndrome (n, n%)	
Anterior cerebral artery	9 (18)
Middle cerebral artery	36 (72)
Posterior cerebral artery	3 (6)
Lacunar syndrome	2 (4)
Side of cerebrovascular accident (n, n%)	
Left	26 (52)
Right	24 (48)

M: males; F: females; BMI: body mass index; SBP: systolic blood pressure; DBP; diastolic blood pressure; MMSE: mini-mental state examination; n: number; %: percentage.

**Table 2:** Fatigue and sleep characteristics of the participants.

Variable	Mean ± SD
FSS	3.82 ± 1.71
ESS	11.46 ± 5.87
ISI	11.78 ± 6.94
PSQI	7.58 ± 3.64
FOSQ	16.60 ± 2.98

FSS: fatigue severity scale; ESS: Epworth sleepiness scale; ISI: insomnia severity index; PSQI: Pittsburg sleep quality index; FOSQ: functional outcome sleep questionnaire.

**Table 3:** Correlation between fatigue and sleep in patients with subacute stroke.

Variables	FSS	
	ρ	*p*
ESS	0.419	0.002
ISI	0.719	0.000
PSQI	0.586	0.000
FOSQ	−0.516	0.000

FSS: fatigue severity scale; ESS: Epworth sleepiness scale; ISI: insomnia severity index; PSQI: Pittsburg sleep quality index; FOSQ: functional outcome sleep questionnaire.

**Table 4:** The mediating role of sleep deficit-related functional loss (FOSQ score) as a mediator in the effect of the sleep quality (PSQI score) on the severity of fatigue (FSS score).

Independent Variable	Outcome Variable	*β*	*b*	SE	95% Bootstrapping CI		*p* Value
					LL	UL	
Sleep Quality	Sleep deficit-related functional loss	−0.46	−0.37	0.10	−0.58	−0.16	<0.001
Sleep deficit-related functional loss	Severity of fatigue	−0.31	−0.18	0.07	−0.32	−0.03	0.016
Sleep quality (direct effect)	Severity of fatigue	0.46	0.22	0.06	0.10	0.33	<0.001
**Types of Effect**		***b***		**SE**	**95% Bootstrapping CI**		***p* Value**
					**LL**	**UL**	
Total effect		0.28		0.05	0.17	0.39	<0.001
Indirect effect		0.07		0.03	0.01	0.13	-

*β*: standardized coefficient; b: unstandardized coefficient; SE: standard error; CI: confidence interval; LL: lower limit; UL: upper limit. FOSQ: functional outcomes of sleep questionnaire; PSQI: Pittsburg sleep quality index; FSS: fatigue severity scale.

**Table 5:** The mediating role of sleep deficit-related functional loss (FOSQ score) as a mediator in the effect of the severity of insomnia (ISI score) on the severity of fatigue (FSS score).

Independent Variable	Outcome Variable	*β*	*b*	SE	95% Bootstrapping CI		*p* Value
					LL	UL	
Severity of insomnia	Sleep deficit-related functional loss	−0.46	−0.20	0.05	−0.31	−0.09	<0.001
Sleep deficit-related functional loss	Severity of fatigue	−0.24	−0.14	0.06	−0.26	−0.01	0.036
Severity of insomnia (direct effect)	Severity of fatigue	0.60	0.15	0.03	0.10	0.20	<0.001
**Types of Effect**		***b***		**SE**	**95% Bootstrapping CI**		***p* Value**
					**LL**	**UL**	
Total effect		0.18		0.02	0.13	0.23	<0.001
Indirect effect		0.03		0.02	0.002	0.06	-

*β*: standardized coefficient; b: unstandardized coefficient; SE: standard error; CI: confidence interval; LL: lower limit; UL: upper limit. FOSQ: functional outcomes of sleep questionnaire; ISI: insomnia severity index; FSS: fatigue severity scale.

**Table 6:** The mediating role of sleep deficit-related functional loss (FOSQ score) as a mediator in the effect of daytime sleepiness (ESS score) on the severity of fatigue (FSS score).

Independent Variable	Outcome Variable	*β*	*b*	SE	95% Bootstrapping CI		*p* Value
					LL	UL	
Daytime sleepiness	Sleep deficit-related functional loss	−0.45	−0.23	0.06	−0.36	−0.10	<0.001
Sleep deficit-related functional loss	Severity of fatigue	−0.41	−0.24	0.08	−0.39	−0.08	0.016
Daytime sleepiness (direct effect)	Severity of fatigue	0.23	0.07	0.04	−0.01	0.15	0.092
**Types of Effect**		***b***		**SE**	**95% Bootstrapping CI**		***p* Value**
					**LL**	**UL**	
Total effect		0.12		0.04	0.05	0.20	0.002
Indirect effect		0.05		0.02	0.01	0.10	-

*β*: standardized coefficient; b: unstandardized coefficient; SE: standard error; CI: confidence interval; LL: lower limit; UL: upper limit. FOSQ: functional outcomes of sleep questionnaire; ESS: Epworth sleepiness scale; FSS: fatigue severity scale.

## Data Availability

The data associated with the paper are not publicly available but are available from the corresponding author on reasonable request.

## Abbreviations

PSF	Post-stroke fatigue
SFS	Sleepiness-related functional status
PSQI	Pittsburgh sleep quality index
ESS	Epworth sleepiness scale
ISI	Insomnia severity index
FOSQ	Functional outcome of the sleep questionnaire
FSS	Fatigue severity scale
